# Predation on Multiple Trophic Levels Shapes the Evolution of Pathogen Virulence

**DOI:** 10.1371/journal.pone.0006761

**Published:** 2009-08-25

**Authors:** Ville-Petri Friman, Carita Lindstedt, Teppo Hiltunen, Jouni Laakso, Johanna Mappes

**Affiliations:** 1 Department of Biological and Environmental Science, Centre of Excellence in Evolutionary Research, University of Jyväskylä, Jyväskylä, Finland; 2 Department of Biological and Environmental Science, University of Helsinki, Helsinki, Finland; University of Hyderabad, India

## Abstract

The pathogen virulence is traditionally thought to co-evolve as a result of reciprocal selection with its host organism. In natural communities, pathogens and hosts are typically embedded within a web of interactions with other species, which could affect indirectly the pathogen virulence and host immunity through trade-offs. Here we show that selection by predation can affect both pathogen virulence and host immune defence. Exposing opportunistic bacterial pathogen *Serratia marcescens* to predation by protozoan *Tetrahymena thermophila* decreased its virulence when measured as host moth *Parasemia plantaginis* survival. This was probably because the bacterial anti-predatory traits were traded off with bacterial virulence factors, such as motility or resource use efficiency. However, the host survival depended also on its allocation to warning signal that is used against avian predation. When infected with most virulent ancestral bacterial strain, host larvae with a small warning signal survived better than those with an effective large signal. This suggests that larval immune defence could be traded off with effective defence against bird predators. However, the signal size had no effect on larval survival when less virulent control or evolved strains were used for infection suggesting that anti-predatory defence against avian predators, might be less constrained when the invading pathogen is rather low in virulence. Our results demonstrate that predation can be important indirect driver of the evolution of both pathogen virulence and host immunity in communities with multiple species interactions. Thus, the pathogen virulence should be viewed as a result of both past evolutionary history, and current ecological interactions.

## Introduction

The pathogen virulence has been traditionally thought to co-evolve in reciprocal selection with its host organism [1]. However, in nature pathogens and their hosts are typically embedded within a web of interactions with other species, which could affect indirectly the evolution of pathogen virulence and host immunity [2]-[4]. For example, predation could increase or decrease the prevalence of infectious diseases depending on how it affects the frequency of infected individuals or high-quality hosts in the population [2], [4]. Moreover, recent findings suggest that predation could affect also directly the pathogen virulence (ability to harm host) and host immunity through trade-offs or positive genetic correlations with traits connected to anti-predatory defence [5]-[7]. Therefore, multi-trophic-level predation could be important selective force affecting the evolution of diseases in natural communities [2]. Yet, experimental studies where the evolutionary consequences of predation on both pathogen virulence and host immunity had been tested simultaneously are rare.

Protozoan predation could increase the pathogen virulence because bacterial defensive adaptations might also have a significant role in bacterial persistence and virulence [6], [8]. For example, protozoa could be important for the enrichment of potentially more pathogenic, biofilm-forming *Vibrio cholerae* strains, which is the principal cause for cholera epidemics [9]. In addition, the survival and successful replication of bacteria inside the protozoan cells have probably gave rise to several facultative and obligate intracellular pathogens, such as *Listeria*, *Rickettsia*, *Mycobacterium*, *Legionella* and *Chlamydia* [10] because the amoebae and macrophages share analogous phagocytic mechanisms, e.g. prey recognition by cell surface receptors [11], prey killing by oxygen radicals [12] and similar digestive enzymes [13].

**Figure 1:**
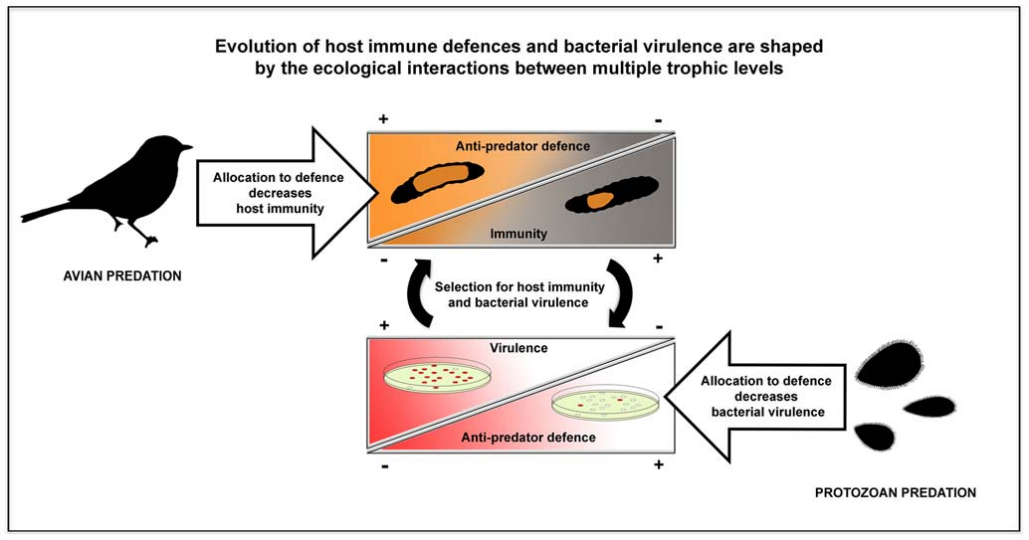
The effect of predation on the evolution of host-pathogen interaction through trade-offs.

Alternatively, protozoan predation could also lead to a decrease in bacterial virulence if increased allocation to anti-predatory traits is traded off with virulence factors of the pathogen. Surprisingly, this hypothesis has not been tested empirically yet, even though previous research suggests that allocation to traits connected to both defence and virulence can be costly [14], [15]. For example, predation by protozoan *Tetrahymena thermophila* has been shown to cause a rapid evolutionary increase in the anti-predator defence of an opportunistic bacterium pathogen *Serratia marcescens* [15]. However, predation also decreased *S. marcescens'* ability to use resources efficiently and decreased the synthesis of the red pigment prodigiosin [15], which has been linked to the expression of several virulence factors in this pathogen [16] (Fig. 1). Therefore, protozoan predation could lead to an evolutionary increase or decrease in bacterial virulence depending on which bacterial defensive traits are selected for, and how these traits are correlated with bacterial virulence factors.

Similarly, predation could affect directly the strength of the host immune system because the resources are often limited and anti-predatory defence and immunity incur costs [5], [17]-[20]. In addition, anti-predatory defences can be traded off with traits affecting host immunity [5], [21], [22]. For example, Rigby and Jokela [5] found that high investment in defence against predators increased freshwater snail's (*Lymnaea stagnalis*) susceptibility to pathogens. Thus, increasing allocation to defence against predation could have indirect costs of reduced host immunocompetence [5]. Interestingly, this kind trade-offs has been shown to play important role in determining the structure of natural communities [7].

To study how selection by predation affects bacterial virulence and host immune defence, three different strains of the ubiquitous bacterium *S. marcescens* with different evolutionary histories were used to infect *Parasemia plantaginis Arctiid* host moth larvae from two selection lines that differed in anti-predatory defence against avian predators. The bacterial strains included an ancestor *S. marcescens* strain (ATCC strain #13880) and two ancestor-derived strains which had been let to evolve in the absence (control strain) or in the presence (evolved strain) of protozoan predator, *Tetrahymena thermophila*, for 14 weeks [15]. All three bacterial strains consisted of a mixture of randomly isolated clones (see methods). Few potential virulence factors of every bacterial strain were also measured (motility and diversity). The host selection lines were artificially selected to have a small (more melanic) or a large (less melanic) orange patch expressed on an otherwise black body [23]. This warning signal is used to indicate unprofitability to bird predators and acts thus as anti-predatory defence [24], [25] (Fig. 1). The bacteria in the genus *Serratia* are common pathogens of many insects including the Lepidoptera [26] and therefore these study species can also potentially encounter in the wild. In the infection experiment, three host groups within both selection lines were infected with one of the tree bacterial strains. In addition, sterilized water was injected into the fourth groups of larvae to control the physical damage caused by the injection itself. Larval survival was monitored for 72 h from infection three times per day by scoring the larvae as dead or alive.

On the basis of previous experiment, we hypothesied that protozoan predation would lead to decreased pathogen virulence due to trade-off with anti-predatory and other life-history traits (e.g. resource use ability, [15]). Similarly, host allocation to a large warning signal, i.e strong defence against avian predators could lead to decreased immune defence through reduction in the amount of larval cuticular melanin (Fig. 1). This is because the black, melanin-based pigment is known to correlate with higher phenoloxidase enzyme activity, which is an important part of the humoral immune response cascade in insects [27], [28].

## Results

**Figure 2:**
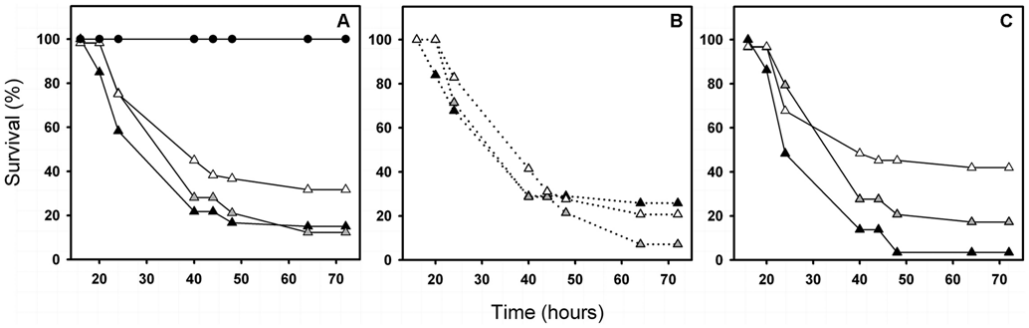
The survival of host larvae infected with bacterial strains differing in their evolutionary histories. Panel a: survival (%) of *P. plantaginis* moth larvae when infected with ancestor (black triangles), control (grey triangles), and evolved (white triangles) strains of the bacteria *S. marcescens*. The straight line (black circles) denotes the survival of control larvae injected with sterilized water. Survival (%) of *P. plantaginis* moth larvae within the small, panel b, and large, panel c, warning signal lines.

When the larval survival was analysed over the host signal lines, ancestor and control *S. marcescens* strains decreased the larval survival compared to larvae infected with the *S. marcescens* strain evolved in the presence of predators (ancestor vs. evolved, Chi-Square = 7.32, *P*<0.007, control vs. evolved, Chi-Square = 3.87, *P*<0.049 and control vs. ancestor, Chi-Square = 1.17, *P* = 0.279, Fig. 2a).

The larval survival did not differ between signal lines (Chi-Square = 0.23, *P* = 0.627). However, the bacterial strains had different effects on larval survival when analysed within the large and small signal lines separately (signal line set as a stratified factor in the analysis). Within the small warning signal line, all the bacterial strains had similar effects on larval survival (all *P*>0.05, Fig. 2b). In contrast, within the large signal line, the larval survival was higher with control and evolved strains, compared to the ancestor strain (control vs. ancestor: Chi-Square = 6.47, *P* = 0.011, evolved vs. ancestor: Chi-Square = 11.47, *P* = 0.001, Fig. 2c).

The larval survival was analysed also within different bacterial treatments (bacterial treatment set as stratified factor in the survival analysis). When the most virulent ancestor strain was used for infection, the larvae with a small warning signal had higher survival compared to larvae with a large warning signal (Chi-Square = 4.85, *P* = 0.028, Fig. 2b and c). However, signal line had no effect on larval survival when control (Chi-Square = 0.54, *P* = 0.459, Fig. 2b and c) or evolved strain was used for infection (Chi-square = 1.033, *P* = 0.309, and also with Cox-regression analysis method, Wald statistics = 4.76, *P* = 0.092, Fig. 2b and c). Damage caused by the injection alone was negligible as none of the larvae died when injected with sterilized water (Fig. 2a). In addition, allocation to a large warning signal did not decrease the encapsulation response of the larvae (small vs. large signal line, F_1, 172_ = 0.02, *P* = 0.882).

**Figure 3:**
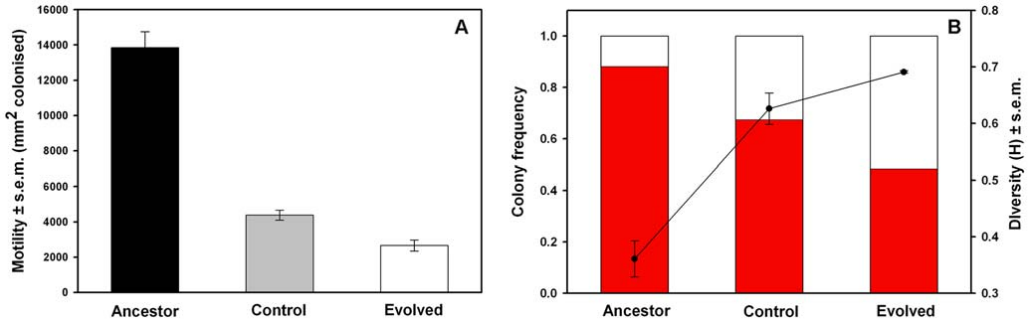
The mean motility (panel a) and diversity (panel b) of different bacterial strains. In panel a, ancestor versus control strain (*P* = 0.001), ancestor versus evolved strain (*P*<0.001) and control versus evolved strain (*P* = 0.014), N = 3 for every strain. In panel b, the mean colony frequencies (bars) and diversity (line) of different bacterial strains determined on the basis of the synthesis of red pigment, prodigiosin.

The motility of control and evolved bacterial strains was considerably lower compared to ancestor strain (t-test for difference in both cases *P*<0.001, Fig. 3a). Yet, the motility of evolved bacterial strain was lowest (t-test for difference between control and evolved strain, *P* = 0.014, Fig. 3a). The frequency of red pigment expressing bacterial clones was highest with the ancestor, intermediate with the control, and lowest with the evolved bacterial strain (main effect of bacterial strain, F_1, 8_ = 62.69, *P*<0.001, in all pairwise comparisons *P* = 0.002 or smaller, Fig. 3b). Conversely, the frequency of non-pigmented bacterial clones was highest with the evolved, intermediate with the control, and lowest with the ancestor bacterial strain (main effect of bacterial strain, F_1, 8_ = 62.69, *P*<0.001, in all pairwise comparisons *P* = 0.005 or smaller, Fig. 3b). The diversity was lowest with the ancestor strain but did not differ between control and evolved strains (main effect of bacterial strain, F_1, 8_ = 50.83, *P*<0.001, ancestor vs. control or evolved strain *P*<0.001, control vs. evolved strain, *P* = 0.11, Fig. 3b).

## Discussion

We found that increased allocation to defence against protozoan predation decreased the *S. marcescens'* virulence (Fig. 2a). The bacterial strains' effect on host survival depended also on the host allocation to warning signal used for defence against avian predation: all the bacterial strains had similar effects on larval survival within the small warning signal (Fig. 2b), while the larval survival was higher with control and evolved strains compared to the ancestor strain within the large signal line (Fig. 2c). This suggests that a pathogen's ability to cause infections does not only depend on its own past evolutionary history, but is also affected by the genetic background of its host.

When the effects of bacterial strains were compared between the signal lines, and when the most virulent ancestral strain was used for infection, the larvae with small warning signal had higher survival compared to larvae with large warning signal (Fig. 2a and b). This suggests that allocation to effective defence against bird predators (i.e. larger warning signal size) can trade off with immune defence against pathogenic bacteria. However, the signal size had no effect on larval survival when less virulent control or evolved strains were used for infection (Fig. 2b and c). Thus, allocation to effective warning signal, i.e. more effective anti-predatory defence against visual predators, might be less constrained when the invading pathogen is rather low in virulence (Fig. 2c).

Protozoan-driven evolutionary decrease in *S. marcescens'* virulence could be explained by decreased motility or resource use ability [15]. Decreased motility has been shown to be connected to the decreased virulence of *Campylobacter jejunum* in piglets [29] and that of Db1140 *S. marcescens* strain in *C. elegans* [30]. Motility could for example affect the bacterial ability to reach favourable habitats within the host [14]. We observed that protozoan predation decreased the motility of *S. marcescens* most relative to the ancestor strain (Fig. 3a). A decrease in bacterial motility, caused probably by down-regulation of flagellum synthesis or other factors related to bacterial motility [31], [32], could have reduced the predator encounter rate leading to more defensive but less virulent bacteria [33], [34]. Since the motility of the control strain was lower relative to the ancestor, but higher relative to the evolved strain, demonstrates that selection by laboratory conditions could not alone select for decreased virulence in *S. marcescens* (Fig. 3a). Replication of the infection experiment with another host, greater wax moth (*Galleria mellonella*), gave consistent results adding more support to view that mere laboratory conditions can decrease the *S. marcescens* virulence but the exposure to protozoan predation decreases the *S. marcescens* virulence most (Friman & Mikonranta, *unpublished*, Figure S1).

Another explanation for the decreased virulence in addition to motility could lie in the predator-induced decrease in prey resource use ability [15]; the less virulent *S. marcescens* could simply be inefficient in obtaining resources within the host, leading to poorer reproduction, and thus a less harmful infection. This hypothesis is supported by the fact that the frequency of white *S. marcescens* clones that were poor at using resources [15] was highest with the evolved strain (Fig. 3b).

In addition, infection with mixture of clones instead of a single clone can affect the bacterial virulence indirectly through competitive or co-operative interactions between different bacterial clones [35], [36]. For example, if bacteria are mainly competing for resources within their host, pathogen diversity is expected to increase virulence through efficient resource use and fast host exploitation [37]. However, if bacteria co-operate, i.e. use exoenzymes to extract nutrients, high relatedness (low diversity) is expected to lead highest resource use and thus also highest virulence [38]. Our data supports the former hypothesis because highest virulence was attained with least diverse mixture of clones, i.e. with the ancestor strain (Fig. 3b). In the case of *S. marcescens*, production of iron scavenging siderophores could be one possible form of co-operation affecting also to its virulence [39]. However, to assess these potential explanations in more detail, infection experiments based on individual clones are needed to characterise the genetic correlations between the traits connected to defence against predation and virulence in *S. marcescens*.

We found that a small warning signal, i.e. more melanic colouration of larvae, was positively linked with defence against the most virulent ancestor *S. marcescens* strain (Fig. 2b–c). However, the possible costs of producing melanin could have counterbalanced the immunological benefits of more melanic larvae when infected with less pathogenic bacteria [24]. Previous studies have also shown that the larvae with large warning signal are faster at growing [40], [24] and most likely explanation for this is that they simply consume more food. This could also help them to boost their immune system because many plants contain toxins, which can be used for example to fight against parasites [41]. Therefore, it is possible that larvae with large warning singal were able to compensate their immune system by sequestering plant toxins more efficiently after and during the infection (food plant, *Taraxacum officinale*, was available throughout the infection experiment).

In general, *P. plantaginis* larvae seemed to allocate more on defence against parasitoids than pathogens. The amount of cuticular melanin did not affect the strength of the larval encapsulation response, which is generally used to describe the immunocompetence of insects against natural parasitoids [27], [42], [43]. Thus, effective defence against macroparasites could have been important for *P. plantaginis* in its past evolutionary history because allocation to an effective warning signal decreased only its resistance against bacterial pathogens. However, more detailed immunological measures are needed to fully understand the linkage between insect cuticular melanin and immune defence against parasites and bacterial pathogens.

Based on predators' learning efficiency, selection is assumed to lead to uniformity and conspicuousness in signal expression thereby decreasing the variation in signal size [25], [44]. However, variation in warning signal expression is common, which suggests that the strength and direction of selection on signal size could vary spatially and temporally [24]. We propose that the observed variation in warning signal pattern of *P. plantaginis* larvae [24] could be partly explained with contrasting selection by avian predators and bacterial pathogens from different trophic levels. Thus, large warning signal size could be favoured when birds are the main cause of larval mortality [25] and the pathogens are rather harmless. Conversely, when the risk of bacterial infection is high (e.g. during the winter hibernation period), larvae with small warning signal, and better immune defence could have advantage. Most importantly, our experiment shows that the pathogen success is not only dependent on its own evolutionary history but is also hugely affected by the host genotype.

There is currently considerable knowledge about the genetic properties and mechanisms that are essential for many pathogenic bacteria to be able to colonise and infect their hosts [42], [45]-[47]. At the same time, relatively little is known about the selective agents and environmental conditions that trigger harmless bacteria to quickly evolve into disease-causing pathogens [1]. Here we show that protozoan predation can decrease the virulence of opportunistic bacterial pathogen *S. marcescens*. Moreover, the host immune defences can evolve indirectly in response to other species interactions, such as predation, which can further affect the success of pathogens. These results demonstrate that virulence is a function of both past evolutionary histories and present ecological interactions of hosts and pathogens. Thus, in order to understand the emergence and dynamics of diseases it could be necessary to understand how evolution affects the pathogen's ability to cause diseases and the host's ability to resist infections in communities with multiple species interactions. This could be achieved by bringing the principles of community ecology, evolutionary theory and host-pathogen epidemiology together.

## Materials and Methods

### Bacterial strains and infection of larvae

The bacterial clones of evolved and control strains were originally isolated from two treatments used in the previous experiment [15] where bacteria were cultured in the absence or presence of protozoa *Tetrahymena thermophila* (ATCC strain #30008) for 14 weeks (approximately 2500 bacterial generations). In both of these treatments, bacteria were originally the same as the ancestor *S. marcescens* strain received from the American Type Culture collection (ATCC strain #13880), which was chosen as the third bacterial strain. All bacterial strains comprised a mixture of 48 randomly isolated clones (four microcosm replicates were used per bacterial strain and 12 clones were isolated randomly per replicate). With ancestral strain, all clones were isolated from one agar plate. The ubiquitous bacteria *S. marcescens* can be found in both aquatic and soil ecosystems, and have an extremely broad host range including plants, nematodes, insects and mammals [26], [45]-[47]. Bacterial strains were first cultivated at 25°C on NB agar plates (containing 2.5 g of yeast extract, 10 g of nutrient broth and 15 g of agar in one litre of dH_2_O). After 48h of growth, bacterial colonies were inoculated in phosphate buffer [15] and diluted to optical densities containing the same number of bacterial cells (colony forming units, main effect of bacterial strain, F_1, 8_ = 0.129, *P* = 0.881). Thereafter, the strains were divided to aliquots, mixed with glycerol and stored at -80°C for later use. Before infection, aliquots of all strains were thawed for 1 h at 25°C. Five µl of well-mixed bacterial solution (approximately 1.66*10^6^ bacterial cells), or sterilized water for the controls, were injected between the second and third segments of larvae with a 10 µl Hamilton syringe. A total of 232 larvae were injected during the experiment: 55 control larvae injected with water, 60 larvae with the ancestor *S. marcescens* strain, 60 larvae with evolved *S. marcescens* strain, and 57 larvae with control *S. marcescens* strain. Infection took place over 6 consecutive days under constant laboratory conditions. Larval survival was not affected by the infection day, F_5, 231_ = 0.4, *P* = 0.847. Before infection, all larvae were weighed (only larvae between 90 and 160 mg were used) and assigned to four groups (per signal line) with approximately the same mean weight to exclude possible condition dependent effects (larval weight between the bacterial treatments had no effect on survival, F_3, 230_ = 0.96, *P* = 0.412, mean weight±S.E. for larvae infected with water: 114.36±2.37 mg, the ancestor strain: 116.6±2.27 mg, the evolved strain: 111.56±2.21 mg, or with the control *S. marcescens* strain: 113.12±1.91 mg.). Each bacterial treatment group consisted of, on average, 58 larvae; 29 with a small and 29 with a large signal (see below). Infection experiment was also replicated with wax moth larvae (*Galleria mellonella*) for each bacterial strain (N = 12) as explained above with the exception that the amount of infected bacterial cells was considerably lower (approximately 10-30 bacterial cells). The injection method has been criticized because it bypasses the entry of microbes through natural routes of infection, e.g. orally [45]. However, bacteria also access hemocoel directly through breaching the cuticle [47] and both infection methods (injection or oral ingestion) have been used with *S. marcescens* to infect a wide range of insect hosts [46], [47].

### Trait measurements of the bacterial strains

Bacterial motility assays were done by stabbing trace amount (2 µl) of each bacterial strain on the centre of semi-fluid NB agar plates containing 0.7% of agar with sterile plastic loops (VWR). The motility of strains was determined as the area (mm^2^) bacteria were able to colonise on the agar plates in 24h (N = 3 for every strain). The frequencies of red (prodigiosin pigment expressing) and white (non-pigmented) bacterial clones were counted from 3 replicate plates for each bacterial strain. The bacterial diversity was estimated as Shannon diversity index on the basis of red and white colony frequencies.

### Host selection lines differing in anti-predatory defence

Selection lines on the extremes of warning signal size in *P. plantaginis* were established in 2004. The larvae were reared under controlled laboratory conditions: temperature, rearing density and food resource (dandelion, *Taraxacum* sp.) were kept constant. Fifty-one families were used to obtain selection lines for divergent phenotypes (for large and small orange signals) by applying a truncated family selection protocol [23]. In other words, individuals with large (proportion of orange over 46% of larvae, i.e.>5 orange segments) and small (proportion of orange less than 31% of larvae, i.e.<5 orange segments) signals were selected and then crossed within the selection lines for several generations. After the 7^th^ generation of selection, the size of the warning signal (proportion of the orange signal in relation to the whole body) was on average 30±0.1% within small, and 52±0.1% within large, selection lines (one-way Anova, F_1, 230_ = 209, *P*<0.001). Neither larval weight nor length differed between selection lines confirming that signal sizes were not a result of differences in body size (one-way ANOVA for weight and length: F_1, 230_ = 195, *P* = 0.659 and F_1, 230_ = 0.45, *P* = 0.502, respectively).

### Encapsulation assessment

Encapsulation reaction is a general response to foreign intrusions in insects [27], [43]. The encapsulation response of all larvae was measured before the bacterial injection. Larvae were anaesthetized with CO_2_, after which a small nylon implant was inserted inside the larvae between the second and the third segments. The immune system of the larvae was allowed to react for 5 hours. Subsequently, the implant was removed, dried and photographed under a microscope with 10×magnification with a Panasonic wv-CL702 video recorder. The mean grey value of the implant was measured with ImagePro Plus 4.0 (Media Cybernetics) on 1 mm of the implant, measured from the end implanted inside the larva. The grey value of the background was subtracted from the grey value of the implant to correct for any variation in lighting during photography. Higher grey values (darker implant) indicated a stronger encapsulation response.

### Statistical analysis

Cox-regression model was built to test if the weight of the larvae before injection was a significant covariate in the model. Due to high insignificance (*P* = 0.638 and coefficient 1.005), the larval weight was omitted from the final analysis and Kaplan-Meier survival analysis and Log-rank statistics were used in analysis. The main effects of bacterial treatment and signal line were analysed first after the effect of bacterial treatment within the signal line and the effect of signal line within the bacterial treatments were analysed using stratification. The Right censoring method was used to include the larvae that did not die within 72 hours in the analysis. The encapsulation ability of larvae was analysed with a one-way ANOVA, and when multiple groups were compared, a two-way ANOVA was used.

## Supporting Information

Figure S1 The survival of alternative host, Wax moth larvae (Galleria mellonella), when infected with ancestor (black triangles), control (grey triangles), and evolved (white triangles) strains of the bacteria S. marcescens. The straight line (black circles) denotes the survival of control larvae injected with sterilized water (ancestor vs. control or evolved strain, P = 0.032 and p<0.001 respectively; control vs. evolved strain, P = 0.05, N = 12 for all groups).
(1.16 MB TIF)
