# Supplementary figures and images for: Predation on Multiple Trophic Levels Shapes the Evolution of Pathogen Virulence
Source: PLoS One. 2009 Aug 25;4(8):e6761. doi: 10.1371/journal.pone.0006761 (PMC2726984; doi:10.1371/journal.pone.0006761)

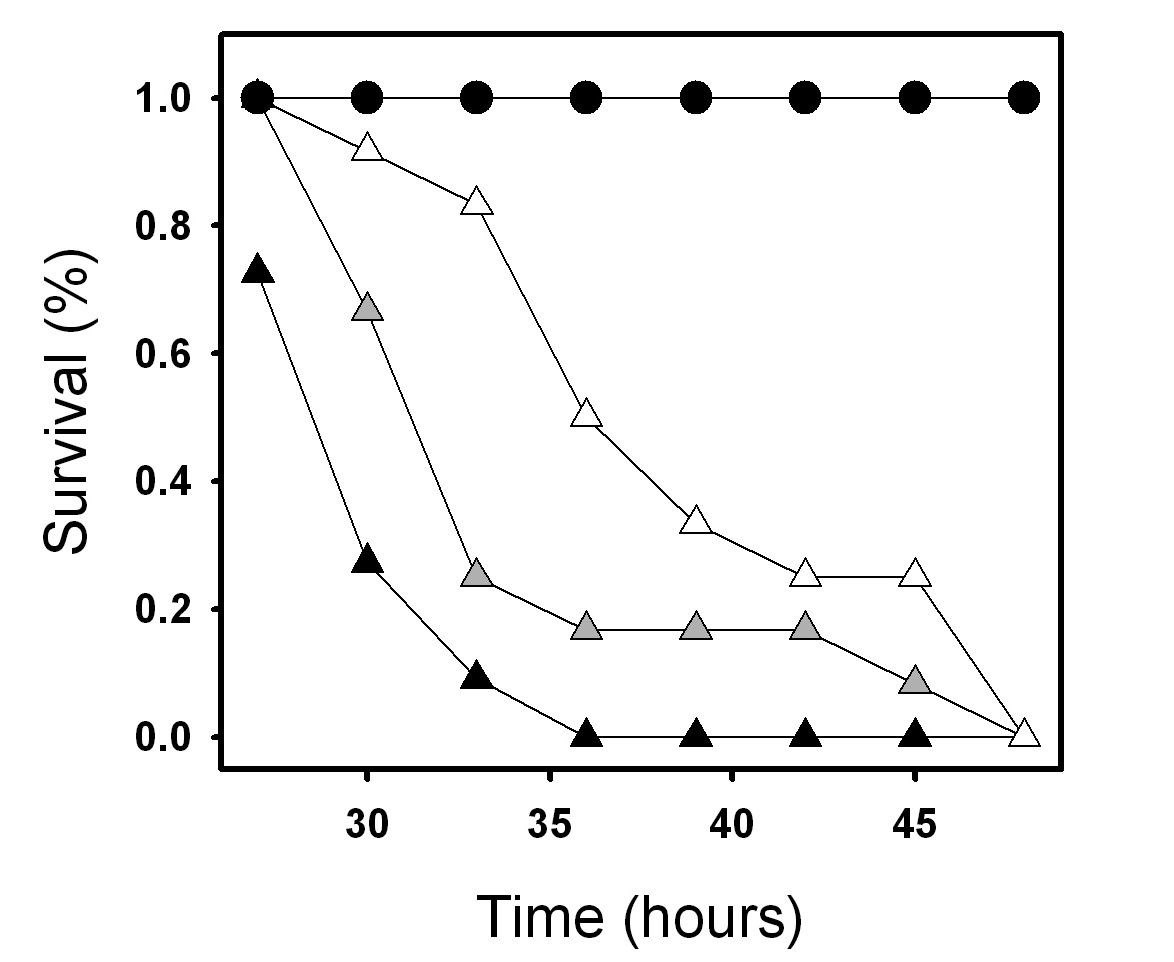

Supplement: Figure S1 — The survival of alternative host, Wax moth larvae (Galleria mellonella), when infected with ancestor (black triangles), control (grey triangles), and evolved (white triangles) strains of the bacteria S. marcescens. The straight line (black circles) denotes the survival of control larvae injected with sterilized water (ancestor vs. control or evolved strain, P = 0.032 and p<0.001 respectively; control vs. evolved strain, P = 0.05, N = 12 for all groups). (1.16 MB TIF) [file pone.0006761.s001.tif]
